# Ultrasonographic predictors of thyroid cancer in Bethesda III and IV thyroid nodules

**DOI:** 10.3389/fendo.2024.1326134

**Published:** 2024-02-09

**Authors:** Ebtihal Y. Alyusuf, Lama Alhmayin, Eman Albasri, Jawaher Enani, Hessa Altuwaijri, Nora Alsomali, Maria A. Arafah, Zahra Alyusuf, Anwar A. Jammah, Aishah A. Ekhzaimy, Ali S. Alzahrani

**Affiliations:** ^1^ Division of Endocrinology, Department of Internal Medicine, College of Medicine, King Saud University, King Saud University Medical City, Riyadh, Saudi Arabia; ^2^ Division of Endocrinology, Department of Internal Medicine, Salmanyia Medical Complex, Government Hospitals, Manama, Bahrain; ^3^ Department of Internal Medicine, College of Medicine, King Saud University, King Saud University Medical City, Riyadh, Saudi Arabia; ^4^ Department of Pathology, College of Medicine, King Saud University, King Saud University Medical City, Riyadh, Saudi Arabia; ^5^ Department of Radiology, Salmanyia Medical Complex, Government Hospitals, Manama, Bahrain; ^6^ Division of Molecular Endocrinology, Department of Molecular Oncology, King Faisal Specialist Hospital and Research Centre, Riyadh, Saudi Arabia; ^7^ Department of Medicine, King Faisal Specialist Hospital and Research Center, Riyadh, Saudi Arabia

**Keywords:** Bethesda III, Bethesda IV, predictors, thyroid cancer, ultrasonography

## Abstract

**Background:**

Bethesda III and IV thyroid nodules continue to be difficult to manage. Although molecular testing may assist in decision-making, it is expensive, not widely available, and not without pitfalls. The objective of this study is to assess whether certain thyroid ultrasonographic features may predict the risk of thyroid cancer in patients with Bethesda III and IV thyroid nodules and be used as additional decision-making tools to complement cytopathological results in deciding on diagnostic thyroidectomy.

**Methods:**

We retrospectively evaluated the ultrasonographic features of Bethesda categories III and IV thyroid nodules in patients who underwent subsequent thyroidectomy. We used the final histopathological examination of the surgical specimens as the gold-standard test and analyzed individual preoperative ultrasonographic features as predictors of malignancy.

**Results:**

Of the 278 patients who were diagnosed with Bethesda III and IV thyroid nodules on fine needle aspiration cytology (FNAC), 111 (39.9%) had thyroid cancer, and 167 (59.9%) exhibited benign nodules. The malignancy rate was higher in patients with Bethesda IV nodules (28/50, 56%) than those with Bethesda III nodules (83/228, 36.4%; *p*=0.016). In univariate analysis, hypoechogenicity (55.6% in malignant vs. 35.3% in benign, *p*=0.006) and calcifications (54.5 in malignant vs. 35.4% in benign, *p*=0.008) were significantly different between the benign and malignant pathology groups, whereas the size of the dominant nodule, number of nodules, irregular borders, taller-than-wide shape, and the presence of lymph nodes were comparable between the two groups. These two ultrasonographic features (hypoechogenicity and calcifications) remained significantly associated with the risk of malignancy in multivariate logistic regression analysis (for hypoechogenicity, *p*=0.014, odds ratio: 2.1, 95% CI:1.0–3.7 and for calcifications, *p*=0.019, odds ratio: 1.98, 95% CI:1.12–3.50). The sensitivity, specificity, positive and negative predictive values, and accuracy were 31.5%, 83%, 55.6%,64.7%, and 62.6%, for hypoechogenicity, respectively and 32.4%, 82%, 54.5%, 67.8%, and 62%, for calcification, respectively.

**Conclusions:**

Hypoechogenicity and calcifications in Bethesda III and IV thyroid nodules are strong predictors of thyroid cancer and associated with a two-fold increased risk of malignancy.

## Introduction

Thyroid nodules are quite common and can be ultrasonographically detected in 50–60% of healthy individuals (1). Although most thyroid nodules are asymptomatic, benign, and do not require surgical treatment, the main clinical challenge in their management is to rule out malignancy. Thyroid ultrasonography and fine-needle aspiration (FNA) biopsy are the primary diagnostic tools for thyroid nodules (2). The American Thyroid Association (ATA) thyroid nodule pattern recognition (2) and the American College of Radiology Thyroid Imaging Reporting and Data Systems (TIRAD) (3) are used to assess the risk of malignancy and the nodular size at which FNA is indicated, whereas the Bethesda classification of thyroid FNA cytology (FNAC) is used for diagnostic classification and therapeutic recommendations (4). The, 2015 ATA nodule pattern recognition classifies nodules into those of high suspicion, with features that include hypoechoic nodules, irregular margin, taller-than-wide shape, and microcalcifications (malignancy risk >70-90%); intermediate suspicion, with features that include soild hypoechogenic nodules but with regular margin (malignancy risk 10-20%); low suspicion nodules that include hyper- or isoechoic nodules and partially cystic nodules with eccentric solid components (malignancy risk 5-10%); very low suspicion nodules that include spongiform nodules and partially cystic nodules without suspicious features (malignancy risk <3%); and benign nodules that include pure cystic lesions (malignancy risk <1%) (2). The TIRAD system is a 5-feature scoring system. These features include composition, echogenicity, shape, margin, and echogenic foci. Each of these features have scores that vary between 0 and 3. The cumulative score of all features is calculated, yielding the final TIRAD score of TR1–TR5 with specific recommendations regarding the size at which FNAC is recommended or size that needs follow up only (3).

Although the management of Bethesda I (non-diagnostic), Bethesda II (benign), Bethesda V (suspicious for malignancy), and Bethesda VI (malignant) thyroid nodules is non-controversial, that of Bethesda III (atypia of undetermined significance/follicular lesion of undetermined significance [AUS/FLUS]) and Bethesda IV (follicular neoplasm/suspicious for a follicular neoplasm) thyroid nodules is more complex due to uncertainties about the risk of malignancy in individual patients (5, 6). Molecular testing improves the estimates of the risk of malignancy and helps in decision-making (7). However, these tests are not widely available, are expensive, and do not eliminate the risk of misdiagnosis. Although diagnostic surgery provides an accurate diagnosis, it is associated with surgical risks, frequently reveals benign pathologies, and may induce long-term hypothyroidism.

Therefore, less expensive and more practical tools are needed to help reduce the rate of unnecessary diagnostic thyroidectomy while decreasing the chance of missing a thyroid cancer diagnosis. The role of thyroid ultrasonography in the initial evaluation of thyroid nodules is well established and is usually performed prior to FNAC to assess the site and characteristics of thyroid nodules and determine whether FNA is needed. However, its role in assessing malignancy risk after FNA has not been well studied. Moreover, most studies included patients with papillary thyroid cancer (PTC), being the most common type of thyroid cancer. However, some studies have investigated the role of preoperative ultrasonographic features of thyroid nodules in estimating the risk of malignancy for other types of thyroid cancer including follicular thyroid neoplasms (8–11), and Medullary thyroid cancer (MTC) (12–14). Although the ultrasonographic features of malignancy (hypoechogenicity, irregular margin, microcalcifications, Taller than wide and solid architecture) remain significant predictors of malignancy, their performance is less obvious in follicular tumors and MTC, tumors that frequently fall in Bethesda III and IV cytology.

In this study, we explored the ultrasonographic features that may predict the risk of malignancy in Bethesda III and Bethesda IV thyroid nodules.

## Materials and methods

### Study design and participants

We reviewed the data of all 787 patients with thyroid nodules who underwent FNAC followed by partial or total thyroidectomy at our center between January, 2015 and January, 2020. Participants with unavailable ultrasonography, FNAC, or surgical histopathological reports were excluded. Since Bethesda III and IV nodules carry a significant risk of malignancy^4^ and since molecular testing is not available in our country, most patients with these types of nodules were sent for diagnostic thyroidectomy unless they refuse or they have significant comorbidities that interfere with surgery. Of these 787 patients, 278 (35.3%) were diagnosed to have Bethesda III or IV thyroid nodules. We analyzed the ultrasonographic features of these thyroid nodules as predictors of malignancy in these two FNAC subgroups. This study was approved by the Institutional Review Board of King Saud University, Riyadh, Saudi Arabia (project ID: E-20-4643). Since the study was retrospective, the request for informed consent was waived.

### Imaging of thyroid nodules

Imaging of thyroid nodules was performed using an ultrasound machine (EPIQ-7, Philips) with a probe frequency of 10–12 MHz. Only two experienced thyroid ultrasonographers (radiologists) reported the results of these images according to the, 2015 ATA management guidelines for pattern recognition (2). We extracted data from these reports including nodule composition (solid or cystic), echogenicity, margin regularity, presence of calcification, shape (taller-than-wide), and extrathyroidal extension.

### FNAC of thyroid nodules

FNAC was performed for patients with nodule size > 1 cm. This was performed in the majority of patients (~70%) by two North-American trained interventional radiologists. Approximately 30% of the FNAC procedures were performed by two endocrinologists certified in thyroid ultrasonography. In this study, all FNAs were performed under ultrasound guidance. A 25-gauge needle, 1.5 inches in length, was connected to a 10cc syringe and used for each FNA pass. Three to six passes were performed for each targeted nodule, without suction. The obtained material was then placed onto two slides, each labeled with the patient’s name, medical file number, and side of the thyroid lobe. One slide was fixed in 95% ethanol for Papanicolaou staining, while the other was air-dried for Diff-Quick staining. The remaining material was rinsed in Cytolyt fixative material to prepare a cell block. Three alcohol-fixed and three air-dried slides, as well as the cell block container for each nodule were transferred to the pathology department within 1–2 hours of the procedure. FNAC was interpreted by one of four cyto-screeners (two were certified by the American Society of Clinical Pathology and the other two by the International Academy of Cytology) and then reviewed and signed by a board-certified cytopathologist. FNAC results were graded using the revised Bethesda system for cytopathology reporting,^4^ which includes six diagnostic categories: (i) non-diagnostic or unsatisfactory, (ii) benign, (iii) AUS/FLUS, (iv) follicular neoplasm or suspicious for a follicular neoplasm, (v) suspicious for malignancy, and (vi) malignant.

### Assessment of ultrasonographic predictors of thyroid cancer

We reviewed the data of all the patients who underwent thyroidectomy between January, 2015 and January, 2020. We excluded patients with any FNA Bethesda class except classes III and IV. We reviewed the preoperative thyroid ultrasonographic reports and analyzed the preoperative ultrasonographic features to predict malignant histopathology and used the final histopathological examination of the surgical specimens as the gold-standard test for malignancy. Ultrasonographic features of thyroid nodules, including size in centimeters, multinodularity, echogenicity, margin, shape, and presence of calcifications or suspicious lymph nodes, were analyzed and compared between the benign and malignant histopathology groups. Demographic and clinical data, including age, sex, body mass index (BMI), and thyroid-stimulating hormone (TSH) levels, were collected from the electronic medical records and evaluated and compared between the two groups.

### Accuracy of ultrasonographic features in diagnosing thyroid malignancy

The performance characteristics, namely, sensitivity, specificity, positive predictive value (PPV), negative predictive value (NPV), and accuracy, of isolated sonographic features in differentiating malignant from benign thyroid nodules in Bethesda III and Bethesda IV nodules and the combination of both Bethesda III and Bethesda IV nodules were calculated. The final nodule status was determined based on surgical pathology results.

### Statistical analyses

Categorical variables were summarized as frequencies and percentages. Normally distributed continuous variables are presented as mean and standard deviation (SD), whereas skewed continuous variables are presented as the median and interquartile range (IQR). Baseline characteristics were compared between the benign and malignant histopathology groups using the T-test for normally distributed continuous variables, Mann-Whitney U test for skewed continuous variables, and Chi-square and Fisher Exact tests for homogeneity for categorical variables. The rates of different ultrasonographic features of thyroid nodules were compared between the benign and malignant histopathology groups using the Chi-square and Fisher exact tests. Multivariate logistic regression analysis was used to assess the association between different ultrasonographic features of thyroid nodules and malignant histopathology. For all these tests, an alpha of 0.05 was set as the cut-off point of statistical significance. The sensitivity, specificity, PPV, NPV, and accuracy for identifying malignant thyroid nodules were calculated. Sensitivity was calculated as the number of true positives/(true positives + false negatives), specificity as the number of true negatives/(true negatives + false positives), PPV as the number of true positives/(true positives + false positives), NPV as the number of true negatives/(true negatives + false negatives), and accuracy as the proportion of true positives + true negatives. All analyses were performed using IBM SPSS version 26 (IBM Corp., Armonk, NY, USA).

## Results

### Baseline characteristics

A total of 787 individuals (132 males, 655 females, with a median age of 46 years and IQR = 37–55 years) underwent partial or total thyroidectomy for thyroid nodules between January, 2015 and January, 2020. All patients underwent diagnostic FNA of the thyroid nodules prior to thyroidectomy. The FNAC results were as follows: 9 (1.1%) Bethesda I, 323 (41%) Bethesda II, 228 (29%) Bethesda III, 50 (6.4%) Bethesda IV, 51 (6.5%) Bethesda V, and 126 (16%) Bethesda VI. Since Bethesda III and IV are the most perplexing clinical categories, we focused our analysis on these two subgroups to define factors associated with a higher risk of malignancy and are, therefore, more compelling for thyroidectomy.

Of the 278 patients with Bethesda classes III and IV, 232 (83.5%) were females and 46 (16.5%) were males with a median age of 45 years (IQR = 36–54 years), a median BMI of 30.1 kg/m^2^ (IQR = 26–34 kg/m^2^), and a median TSH level of 2.0 mU/L (IQR = 1.0–3.0 mU/L). The number of nodules per patient was as follows: a single nodule in 46 patients (16.5%), 2 nodules in 57 patients (20.5%), 3 nodules in 8 patients (2.9%), ≥ 4 nodules in 131 patients (47.1%), and an unclear number in 36 patients (13%). The median size of the dominant nodules was 3 cm (IQR = 2–4 cm). The other ultrasonographic features of the nodules were as follows: 133 nodules (47.8%) were solid, two nodules (0.7%) had irregular borders, 21 nodules (7.6%) were isoechoic, 63 nodules (22.7%) were hypoechoic, 66 nodules (23.7%) exhibited calcifications, and 17 patients (6.1%) exhibited the presence of suspicious lymph nodes (**Table 1**).

**Table 1:** Baseline patient and thyroid nodule characteristics for Bethesda categories III and IV (n = 278)*.

Features	All n = 278
Age, median (IQR), years	45 (36–54)
Sex, n, (%)	
Women	232 (83.5)
Men	46 (16.5)
BMI, median (IQR), kg/m^2^	30.1 (26–34)
TSH, median (IQR), mU/L	2.0 (1.0–3.0)
Nodules number, n, (%)	
Single	46 (16.5)
2	57 (20.5)
3	8 (2.9)
≥ 4	131 (47.1)
Unclear	36 (13)
Size of the dominant nodule, median (IQR), cm	3.0 (2.0–4.0)
Solid, n, (%)	133 (47.8)
Irregular border, n, (%)	2 (0.7)
Echogenicity, n, (%)	
Isoechoic	21 (7.6)
Hypoechoic	63 (22.7)
Calcifications, n, (%)	66 (23.7)
Suspicious lymph nodes, n, (%)	17 (6.1)

IQR, interquartile range; BMI, body mass index; TSH, thyroid-stimulating hormone.

^*^Data are shown as the number of participants (%) or median (IQR), as appropriate.

### Rate of thyroid cancer

Of the 278 patients with Bethesda III and IV nodules, 111 (39.9%) were confirmed as having thyroid cancer, whereas 167 (60.1%) had benign thyroid lesions based on the surgical histopathological examination. The rate of malignancy was significantly higher in Bethesda IV nodules (28/50, 56%) than in Bethesda III nodules (83/228, 36.4%) (p = 0.016). The frequency of thyroid cancer subtypes in Bethesda III and IV nodules are summarized in **Table 2**. The classic subtype of PTC is the most frequent malignant tumor among both groups (26.3% in Bethesda III and 26% in Bethesda IV).

**Table 2:** Thyroid tumor subtypes on final histopathological examination of surgical samples in Bethesda III and IV thyroid nodules^*^.

Tumor subtype	Bethesda III n (%)	Bethesda IV n (%)	Total n (%)
Benign tumor	145 (63.6)	22 (44)	167 (60.1)
Classic papillary thyroid cancer	60 (26.3)	13 (26)	73 (26.2)
Follicular variant papillary thyroid cancer	9 (3.9)	3 (6)	12 (4.3)
Tall cell variant papillary thyroid cancer	1 (0.4)	0	1 (0.4)
Follicular thyroid cancer	6 (2.6)	5 (10)	11 (3.9)
Oncocytic thyroid cancer	5 (2.2)	7 (14)	12 (4.3)
Medullary thyroid cancer	2 (0.9)	0	2 (0.7)
**Total**	228 (100)	50 (100)	278 (100)

*Data are presented in frequency (%).

### Association between thyroid histopathology and various clinical and ultrasonographic characteristics of Bethesda categories III and IV thyroid nodules

Based on a univariate analysis, there was a significant difference in the rates of thyroid nodule hypoechogenicity (31.5% in malignant vs. 16.8% in benign pathology, *p* = 0.006) and calcifications (32.4% in malignant vs. 18% in benign pathology, *p* = 0.008) between the malignant and benign pathology groups. However, the size of the dominant nodule (median 3.0 cm, IQR 2.0-5.0) in malignant vs. (median 3.0 cm, IQR 2.0-5.0) in benign, (*p* = 0.52), the number of nodules (*p* = 0.65), the rate of irregular margins (0.9% in malignant vs. 0.6% in benign, *p* = 1, *p* = 1.0), the rate of taller-than-wide morphology (0% in malignant vs. 0% in benign, *p* = 1.0), and presence of suspicious lymph nodes (5.4% in malignant vs. 6.6% in benign, *p* = 0.88) were comparable between the malignant and benign nodules (**Table 3**). Moreover, patients with malignant and benign histopathology were comparable in terms of age (median 45 years, IQR 36-55) in malignant vs. (median 43 years, IQR 37-53) in benign, (*p* = 0.39), sex (female: 95 [85.6%] in malignant vs. 137 [82%] in benign, *p* = 0.53), BMI (median BMI, 30 kg/m^2^, IQR 26.4-34.1 in malignant vs. 29 kg/m^2^, IQR 25-34 in benign, *p* = 0.46), and TSH level (median TSH level, 2.0 mU/L, IQR 1.0-3.0 mU/L in malignant vs. 2.0 mU/L, IQR 1.0-3.0 mU/L in benign, *p* = 0.98).

**Table 3:** Univariate analysis of the association of various clinical and ultrasonographic features of Bethesda III and IV thyroid nodules and the surgical histopathology (n = 278)^*^.

Variables	Surgical histopathology		P-value†
	Benign n = 167	Malignant n = 111	
Age, median (IQR), years	43 (37-53)	45 (36-55)	0.39
Sex, n, (%)			
Male	30 (18)	16 (14.4)	0.53
Female	137 (82)	95 (85.6)	
BMI, median (IQR), kg/m^2^	29 (25-34)	30 (26.4-34.1)	0.46
TSH, median (IQR), mU/L	2.0 (1.0-3.0)	2.0 (1.0-3.0)	0.98
Nodules number, n, (%)			
Single	28 (16.9)	18 (16.2)	0.65
2	29 (17.5)	28 (25.2)	
3	5 (3)	3 (2.7)	
≥ 4	82 (49.4)	49 (48.1)	
Unclear	22 (13.3)	13 (11.7)	
Size of the dominant nodule, Mean ± SD, cm	2.9 ± 1.7	3.1 ± 1.5	0.27
Solid, n, (%)	77 (55.4)	56 (58.9)	0.69
Irregular margin, n, (%)	1/167 (0.6)	1/111 (0.9)	1.0
Taller than wide, n, (%)	0/111	0/167	1.0
Isoechoic pattern, n, (%)	16/167 (9.6)	5/111 (4.5)	0.18
Hypoechoic pattern, n, (%)	28/167 (16.8)	35/111 (31.5)	0.006
Calcifications, n, (%)	30/167 (18)	36/111 (32.4)	0.008
Suspicious lymph node, n, (%)	11/167 (6.6)	6/111 (5.4)	0.88

BMI, body mass index; TSH, thyroid-stimulating hormone; SD, standard deviation.

^*^Data are shown as mean ± SD or number of participants (%) as appropriate.

†P-values for the differences in surgical histopathology evaluation (benign vs. malignant) were obtained using the t-test, Mann-Whitney U test, Chi-square test, or Fisher exact test, as appropriate.

### Multivariate logistic regression analysis results

Among all ultrasonographic features, hypoechogenicity (odds ratio [OR] = 2.1, 95% confidence interval [CI] = 1.2–3.7, *p* = 0.014) and calcifications (OR = 1.98, 95% CI = 1.12–3.50, *p* = 0.019) remained significantly associated with the risk of malignancy.

### Accuracy of hypoechogenicity and calcifications in diagnosing thyroid malignancy

The diagnostic parameters for nodule hypoechogenicity and calcifications are summarized in **Table 4**. Since these ultrasonographic features are mainly concerned with the increasing risk of diagnosis of malignancy, PPVs of hypoechogenicity are 54.3, 58.8, and 55.6%, for Bethesda III, IV, or both, respectively, and the PPVs for calcification are 54.2, 57, and 54.5%, respectively, for Bethesda III, IV, or both. Other diagnostic parameters are summarized in **Table 4**.

**Table 4:** Sensitivity, specificity, PPV, NPV, and overall accuracy of hypoechogenicity and calcifications in Bethesda III and/or IV thyroid nodules in diagnosing thyroid malignancy.

	Ultrasonographic feature	Sensitivity (%)	Specificity (%)	PPV (%)	NPV (%)	Accuracy (%)
**Bethesda** **III & IV**	Hypoechogenicity	31.5	83	55.6	64.7	62.6
	Calcification	32.4	82	54.5	67.8	62%
**Bethesda** **III only**	Hypoechogenicity	30	85.5	54.3	68.1	65.3
	Calcification	38.6	81.4	54.2	69.8	65.8
**Bethesda** **IV only**	Hypoechogenicity	35.7	68.2	58.8	45.5	50
	Calcification	14.3	86.4	57	44.2	46

PPV, positive predictive value; NPV, negative predictive value.

## Discussion

This study showed that hypoechogenicity and calcifications in the ultrasonographic morphology of Bethesda III and IV thyroid nodules are independent predictors of malignancy. The presence of any of these two ultrasonographic features is associated with a two-fold increase in the risk of thyroid cancer in Bethesda categories III and IV thyroid nodules, a finding that could facilitate the therapeutic plan and justify thyroid surgery for a group of patients with thyroid nodules with these features. Although the sensitivities of hypoechogenicity and calcifications are low for the prediction of malignancy in Bethesda III and IV thyroid nodules, the specificities are quite high, a finding that indicates a better performance of these two features in confirming thyroid malignancy, whereas their absence in the ultrasonographic image does not rule out thyroid malignancy. The moderate levels of sensitivities for hypoechogenicity and calcification were not unexpected and could be explained by the low prevalence of these two ultrasonographic features. Although some studies have shown no association between ultrasonographic features and malignancy in indeterminate thyroid nodules (15, 16), our results are consistent with those reported by many other researchers (17–21). In a meta-analysis performed to evaluate the diagnostic value of thyroid ultrasound for Bethesda III thyroid nodules, Gao et al. found that the presence of any of the suspicious ultrasonographic features (hypoechogenicity, calcifications, irregular margin, taller-than-wide shape, or increase in nodule size during follow-up) as indicators of malignancy was associated with a pooled sensitivity of 0.75 and specificity of 0.48, and the greater the number of suspicious features, the more likely it was a malignancy (17). In addition, our findings are in accordance with those of a study by Li et al., who found that hypoechogenicity and microcalcification, as well as irregular borders and a taller-than-wide shape increased the risk of thyroid cancer in Bethesda III and IV nodules with an odds ratio of 2.02 for hypoechogenicity and 3.21 for microcalcifications (18). However, Hacim et al. found in their observational study that hypoechogenicity, solitary nodules, and solid structures, but not calcifications, were associated with malignancy in Bethesda III, IV, and V thyroid nodules (19). A regional study by Alshahrani et al. reported that hypoechogenicity and microcalcification, in addition to irregular margins and multiple nodules, are highly suspicious features of malignancy in Bethesda III thyroid nodules (20). The vast majority of patients included in these studies were PTC. However, Several retrospective studies have reported the performance of ultrasonographic features of thyroid nodules in predicting malignancy in other subtypes of thyroid cancer. Gao L. et al. identified that penetrating vascularity can help to identify MTC among nodules with low to intermediate suspicion for malignancy (22). Kim S.H. et al. compared 21 MTC with 114 PTC and found that the US features of the two types of thyroid cancer are not that different except that ovoid to round shape was more prevalent in MTC than PTC (12). In another study to identify the ultrasonographic features that differentiate follicular carcinoma from adenoma, the authors reported that follicular carcinomas are characterized by heterogeneous echogenicity, speculated/ill-defined margin, and the presence of calcifications on ultrasound (10). Matrone A. et al. evaluated the ultrasonographic features of thyroid nodules in non-invasive follicular thyroid neoplasm with papillary-like nuclear features (NIFT-P), follicular variant of PTC (FV-PTC), FTC, or follicular adenoma (FA), and found that FV-PTC and FTC are more frequently associated with irregular margins, presence of calcifications, taller than wide shape, and absent halo compared with NIFT-P (8). Sgro’ D. et al. studied the correlation of the histopathological variants of thyroid cancer with the ultrasonographic features and cytology (14). They reported that the presence of microcalcifications, hypoechogenic patterns, and irregular margins, correlated with malignancy and are typical of classic variant PTC, whereas the association of hypoechogenic pattern, irregular margins, and no microcalcifications was more frequent in tall cell subtype PTC than in classic subtype PTC.

The treatment decision for indeterminate (Bethesda III and IV) thyroid nodules poses a great challenge for endocrinologists and endocrine surgeons (23) and the optimal management of this category of thyroid nodules remains unclear. Studies have shown a wide variation in malignancy rates, and the available diagnostic methods have limited ability to differentiate between malignant and benign lesions in these two Bethesda groups. The rates of malignancy in our results (36.4% in Bethesda III and 56% in Bethesda IV) were higher than those originally recommended by the Bethesda system (10–30% for Bethesda III and 25–40% for Bethesda IV) (4), but they concur with those reported by several subsequent international (10–50% in Bethesda III and 25–70% in Bethesda IV) (5, 6, 24–26) and regional studies (14–46% in Bethesda III and 47% in Bethesda IV) (20, 27). The higher rate of malignant pathology in this group of indeterminate thyroid nodules compared to the original rates recommended in the Bethesda system (4) could be related to a more surgical approach in our patients, different population malignancy rates, or referral bias because our hospital is a tertiary care referral center.

Molecular testing is an emerging tool to stratify the malignancy risk in indeterminate thyroid nodules and helps clinicians avoid unnecessary thyroid surgeries by ruling out malignancy (28, 29); however, it is not available in our center or most centers in our region, as is the case in most countries. In a multicenter study that examined ultrasonographic and clinical features for their correlation with malignancy predicted by molecular testing, ultrasonographic features alone did not contribute to predicting cancer risk (30). However, combining molecular testing with sonographic risk stratification improves malignancy prediction (31). Although the cost-effectiveness of molecular testing in patients with indeterminate thyroid nodules has been reported (32), considering the high prevalence of thyroid nodules, including those with indeterminate cytology, the cost of molecular testing can be high, and this is one of the limitations of its use. Molecular testing is expensive and not widely available. Therefore, looking for cheaper, readily available, comparable, or better diagnostic methods for patients with indeterminate cytology is prudent, and our study suggests that the presence of hypoechogenicity and/or calcifications on ultrasonographic examination could improve diagnostic workup and decision-making. This may decrease the rate of unnecessary thyroidectomy but is more likely to increase the yield of diagnostic thyroidectomy when these ultrasonographic features are combined with cytology results.

Our study had some limitations and strengths. The limitations include the retrospective design and lack of randomization and blinding, which may have contributed to selection bias. However, the study’s retrospective nature is more representative of day-to-day practice and may decrease the reporting bias of ultrasonographic features. FNAC was interpreted by two North-American trained cytopathologists, but interobserver variability is a well-known phenomenon in thyroid cytopathology. Similarly, ultrasonographic findings were reported by two North-American-trained radiologists, but interobserver variability is another well-recognized phenomenon in thyroid ultrasonography. However, these limitations are not specific to our study and are inherent in essentially all studies of similar nature. In our practice, we follow the ATA size limit for FNAC (>1 cm). This is different from the size limits of the TIRAD system and we do not know if our study would show similar results if the TIRAD system was followed. Therefore, in practice that depends heavily on the TIRAD system for selection of patients for FNAC, results may vary. Another potential limitation is that patients who may have been selected for surgery were perceived to have higher risk of malignancy and this may potentially induce selection bias. However, our practice has been mostly to perform diagnostic thyroidectomy in the vast majority of patients with Bethesda III and IV because these categories carry a significant risk of malignancy amounting up to 30–40%, and because molecular testing is not available to help decrease the need for surgery. Exceptions to this approach are patient’s preference of not having surgery or the presence of comorbidities that may increase the risk of surgery. Therefore, this approach minimizes selection bias, although it may not totally eliminate it. In addition, the use of surgical histopathology as the gold-standard test for every case is a strength of our study. We believe that further exploration of ultrasonographic features as predictors of malignancy in thyroid nodules is warranted and may prove to be comparable but much cheaper and accessible than molecular testing.

In conclusion, this study showed that the presence of either hypoechogenicity or calcifications in the ultrasonographic features of thyroid nodules in the Bethesda categories III and IV is associated with a two-fold increased risk of malignancy and is a strong indication for thyroid surgery.

## Data availability statement

The raw data supporting the conclusions of this article will be made available by the authors, without undue reservation.

## Ethics statement

The studies involving humans were approved by Institutional Review Board of King Saud University, Riyadh, Saudi Arabia. The studies were conducted in accordance with the local legislation and institutional requirements. Written informed consent for participation was not required from the participants or the participants’ legal guardians/next of kin in accordance with the national legislation and institutional requirements.

## Author contributions

EYA: Data curation, Supervision, Writing – original draft, Writing – review & editing. LA: Data curation, Writing – original draft, Writing – review & editing. EA: Data curation, Writing – original draft, Writing – review & editing. JE: Data curation, Writing – original draft, Writing – review & editing. HA: Data curation, Writing – original draft, Writing – review & editing. NA: Data curation, Writing – original draft, Writing – review & editing. MA: Resources, Writing – review & editing. ZA: Data curation, Writing – review & editing. AJ: Conceptualization, Investigation, Methodology, Project administration, Resources, Supervision, Visualization, Writing – original draft, Writing – review & editing. AE: Conceptualization, Investigation, Methodology, Project administration, Resources, Supervision, Visualization, Writing – original draft, Writing – review & editing. AA: Investigation, Methodology, Project administration, Resources, Supervision, Visualization, Writing – original draft, Writing – review & editing.
